# The D153del Mutation in GNB3 Gene Causes Tissue Specific Signalling Patterns and an Abnormal Renal Morphology in Rge Chickens

**DOI:** 10.1371/journal.pone.0021156

**Published:** 2011-08-22

**Authors:** Hemanth Tummala, Stewart Fleming, Paul M. Hocking, Daniel Wehner, Zahid Naseem, Manir Ali, Christopher F. Inglehearn, Nikolai Zhelev, Douglas H. Lester

**Affiliations:** 1 School of Contemporary Sciences, University of Abertay Dundee, Dundee, Scotland, United Kingdom; 2 The Roslin Institute and Royal (Dick) School of Veterinary Studies, University of Edinburgh, Easter Bush, Midlothian, Scotland, United Kingdom; 3 Section of Ophthalmology and Neuroscience, Leeds Institute of Molecular Medicine, St James's University Hospital, Leeds, United Kingdom; 4 Division of Medical Sciences, Centre for Oncology and Molecular Medicine, College of Medicine Dentistry and Nursing, Ninewells Hospital and Medical School, Dundee, Scotland, United Kingdom; University of Illinois at Chicago, United States of America

## Abstract

**Background:**

The GNB3 gene is expressed in cone but not rod photoreceptors of vertebrates, where it acts as the β transducin subunit in the colour visual transduction process. A naturally occurring mutation ‘D153del’ in the GNB3 gene causes the recessively inherited blinding phenotype retinopathy globe enlarged (rge) disease in chickens. GNB3 is however also expressed in most other vertebrate tissues suggesting that the D153del mutation may exert pathological effects that outlie from eye.

**Principal Findings:**

**** Recombinant studies in COS-7 cells that were transfected with normal and mutant recombinant GNB3 constructs and subjected to cycloheximide chase showed that the mutant GNB3d protein had a much shorter half life compared to normal GNB3. GNB3 codes for the Gβ3 protein subunit that, together with different Gγ and Gα subunits, activates and regulates phosphorylation cascades in different tissues. As expected, the relative levels of cGMP and cAMP secondary messengers and their activated kinases such as MAPK, AKT and GRK2 were also found to be altered significantly in a tissue specific manner in rge chickens. Histochemical analysis on kidney tissue sections, from rge homozygous affected chickens, showed the chickens had enlargement of the glomerular capsule, causing glomerulomegaly and tubulointerstitial inflammation whereas other tissues (brain, heart, liver, pancreas) were unaffected.

**Significance:**

These findings confirm that the D153del mutation in GNB3 gene targets GNB3 protein to early degradation. Lack of GNB3 signalling causes reduced phosphorylation activity of ERK2 and AKT leading to severe pathological phenotypes such as blindness and renal abnormalities in rge chickens.

## Introduction

Heterotrimeric G proteins in the cell serve as molecular switches for important signalling cascades, including those that control heart rate, blood pressure and glucose metabolism and those that mediate the senses of taste, smell, and vision [1]. The heterotrimeric G proteins themselves are activated by G-protein-coupled receptors (GPCRs), which reside in the cell membrane and react to specific external signals, such as light or hormones [2], [3]. G-proteins consist of 3 different subunits, denoted as α, β and γ which are assembled as heterotrimeric complexes under basal state conditions. Sixteen different vertebrate genes have been identified that encode Gα subunits, five genes encode Gβ subunits (GNB1-5) and thirteen genes encode Gγ subunits [1]. Specific combinations of the many different Gα and Gβγ subunits are required for connecting individual receptors to signalling pathways in most cells of the vertebrate body [4]. These three G proteins (Gα, Gβ &Gγ) interact in different combinations to determine the nature of the downstream signal [4]. Following stimulation of an inactive GPCR, by light or ligand, the receptor conformation changes, altering its interaction with all three bound heterotrimeric G proteins [2]. The Gαsubunit is then activated by GTP phosphorylation and subsequently dissociates from the Gβγ dimer, which acts as a single functional unit. Different possible Gβγ dimer combinations suggest functional selectivity by interacting at GPCR interfaces along with effectors of cellular components that are regulated post-translationally [5], [6]. Gβγ dimers provide a great potential for diversity and selectivity, initiating a scaffold of proteins through distinct downstream signalling cascades such as phospholipase C (PLC), phosphoinositide 3Kinase (PI3K) and G-protein receptor kinases (GRK's) [3].

The Gβγ dimer was formerly considered as extraneous to the Gα mediated coupling of GPCRs to downstream signaling effectors. However recent research evidence suggests that it has its own rich set of downstream signaling targets [7]. Recent studies have indicated it has been shown that differential activation of Gβγ dimers alters many downstream signalling pathways that include the mitogen activate protein kinase (MAPK) cascade through RAS pathway in regulating the phosphoproteome [7]–[13]. Extra cellular regulated Kinase (ERK) 1 (MAPK3) and 2 (MAPK1) enzymes of the MAPK cascade are evolutionary conserved in regulating cell signal transduction by connecting cell-surface receptors to critical regulatory targets within cells. These pathways are essential in controlling cell survival, proliferation and apoptosis. The chicken genome only possess the mammalian orthologue of ERK2 [14] suggesting that this gene later duplicated itself and evolved into ERK1 in a mammalian progenitor species, after the divergence of avian species. ERK1/2 activation has distinct role in modulating endocytosis either by sequestering or nonsequestering of the activated GPCR's [15]. Agonist occupied or constitutively activated GPCR's are phosphorylated and desensitized by kinase molecules GRK's, which are evolutionarily conserved and present in both mammals and chickens [Bibr pone.0021156-Larhammar1] [16,17]. GRK2 belongs to second sub family of GRK's, which are specifically regulated by Gβγ signalling upon their binding. These events have previously been shown to phosphorylate and desensitize its associated GPCR by regulating arrestin binding and activating endocytosis pathways [Bibr pone.0021156-Thompson1] [Bibr pone.0021156-Premont1] [18–20]. The diversity in tissue expression of various Gβ and Gγ subunits suggests a role for Gβγ signalling in regulating the traffic of GPCRs on cell membrane.

Inherited variations in genes that alter the structure of Gβ subunits are therefore likely to differentially activate the Gβγ signalling potential to alter both phosphorylation and endocytosis potential of GPCR's, in a timely dependent manner. Alternative splicing of the five genes that encode Gβ subunits introduces even greater potential for the functional diversity of Gβγ dimers. Interestingly a common human variant GNB3s subunit, coded by the 825T allele (OMIM 139130) causes enhanced signalling in downstream targets of GNB3 and the hyper activation of functional G protein signalling pathways [21]. The GNB3 825T allele has a frequency of between 21 to 91% in the different populations that have been studied to date [21]. This allele has been shown to be a significant predisposing factor for common diseases such as Alzheimer's, hypertension, obesity, low birth weight, increased ventricular mass and coronary heart disease [22]. However, Bullido et al. [23] demonstrated a significant increase in both MAPK activity and cAMP levels in HEK-293 cells transfected with recombinant GNB3 825T plasmid constructs, compared to 825C constructs. These results were consistent with observed biochemical changes in the brains of patients with Alzheimer's disease [23]. Despite the interest provoked by studies showing that the enhanced signalling of GNB3s causes an increase in the risk of developing brain disorders, hypertension [24] and coronary heart disease [25] in humans, a definitive understanding of the mechanisms underlying these pathologies has not yet been reached. GNB3 has previously been shown to bind both Gαi and Gαs subunits [26] and, more recently, to interact with specific gamma subunits, which thereby activate different isoforms of the PLC pathway [27].

Interestingly other transcripts have been identified in association with T allele, which give rise to stable protein structures such as GNB3s2 [28]. GNB3s2 is found to be activating the mitogen-activated protein kinase cascade suggesting that it is a biologically active GNB variant, which may play a role in the manifestation of the complex phenotype associated with the 825T-allele. Contrastingly another novel splice variant of GNB3, termed Gbeta3v, which is generated by alternative splicing of parts from intron 9 as a novel exon 10 of the GNB3 gene is found to have no association with the 825T-allele. GNB3v protein form stable dimers with γ subunits but tend not to be biologically active in activating signalling pathways such as PLCβ2 [29]. However, the lack of an appropriate mouse model or the availability of GNB3-genotyped fresh human tissue suitable for biochemical studies has hindered further progress on elucidating the tissue specific pathways affected by GNB3 or its variants.

We previously reported a mutation (D153del) in the GNB3 gene, which causes a recessive, progressive retinal dystrophy known as retinopathy globe enlarged (rge OMIA 2724) in chickens [30]. In rge affected chickens there is a variable degree of vision loss within 24 hours of hatching, with progressive deterioration in vision over the next few weeks, leading to complete blindness after 8 weeks. Unusually for hereditary retinopathies, this vision loss is not the result of photoreceptor loss. The rge chickens do however show a progressive and significant developmental disruption of both rod and cone photoreceptor synaptic terminals [31]. The retinal globe enlargement phenotype observed in rge chickens, has recently been shown to be associated with developmental disruptions of collagen organisation in the cornea [Bibr pone.0021156-MontianiFerreira2] [Bibr pone.0021156-Boote1] [31–33].

Interestingly the highly conserved GNB3 gene is expressed not only in the eye, but also in most other vertebrate tissues [30], with the notable exception of retinal rod cells [Bibr pone.0021156-Peng1] [34,35]. Previous bioinformatic 3D secondary structural modeling of the D153del GNB3 mutation indicated that this mutant structure is unstable and therefore unlikely to be functional [30]. In support of this hypothesis, slot blot experiments have shown a decrease in the amount of immuno-reactive GNB3 protein levels in homozygous affected rge chicken retinas compared to normal retinal tissue [30]. As the RNA expression levels for the D153del GNB3 allele appear to be unaffected [30], this suggests that the D153del mutation results in an unstable GNB3d protein (‘d’ refers to degradation), which is probably rapidly degraded upon synthesis. This is in contrast to the stable normal GNB3, GNB3s, GNB3s2 and GNB3v subunits in humans [Bibr pone.0021156-Siffert3] [Bibr pone.0021156-Rosskopf2] [25,28,29]. The rge chicken expressing mutant GNB3d subunit is therefore a naturally occurring GNB3 knock out model for studying alterations in G protein signaling regulated specifically through Gβγ.

In this study we report the effects of the D153del mutation on the GNB3 protein subunit affecting its cellular localisation, stability and defects involving different secondary messenger levels, which affect phospo-regulatory events in cellular signalling. The retinal specific biochemical changes observed in rge chickens may help to explain the blinding retinopathy globe enlarged phenotype. Similarly the alterations in kidney specific signaling mechanisms in the rge chickens are also likely to underlie the severe renal abnormalities in the kidney sections of these birds

## Results

### Effect of D153del mutation on GNB3 protein expression and localisation

**Figure 1:**
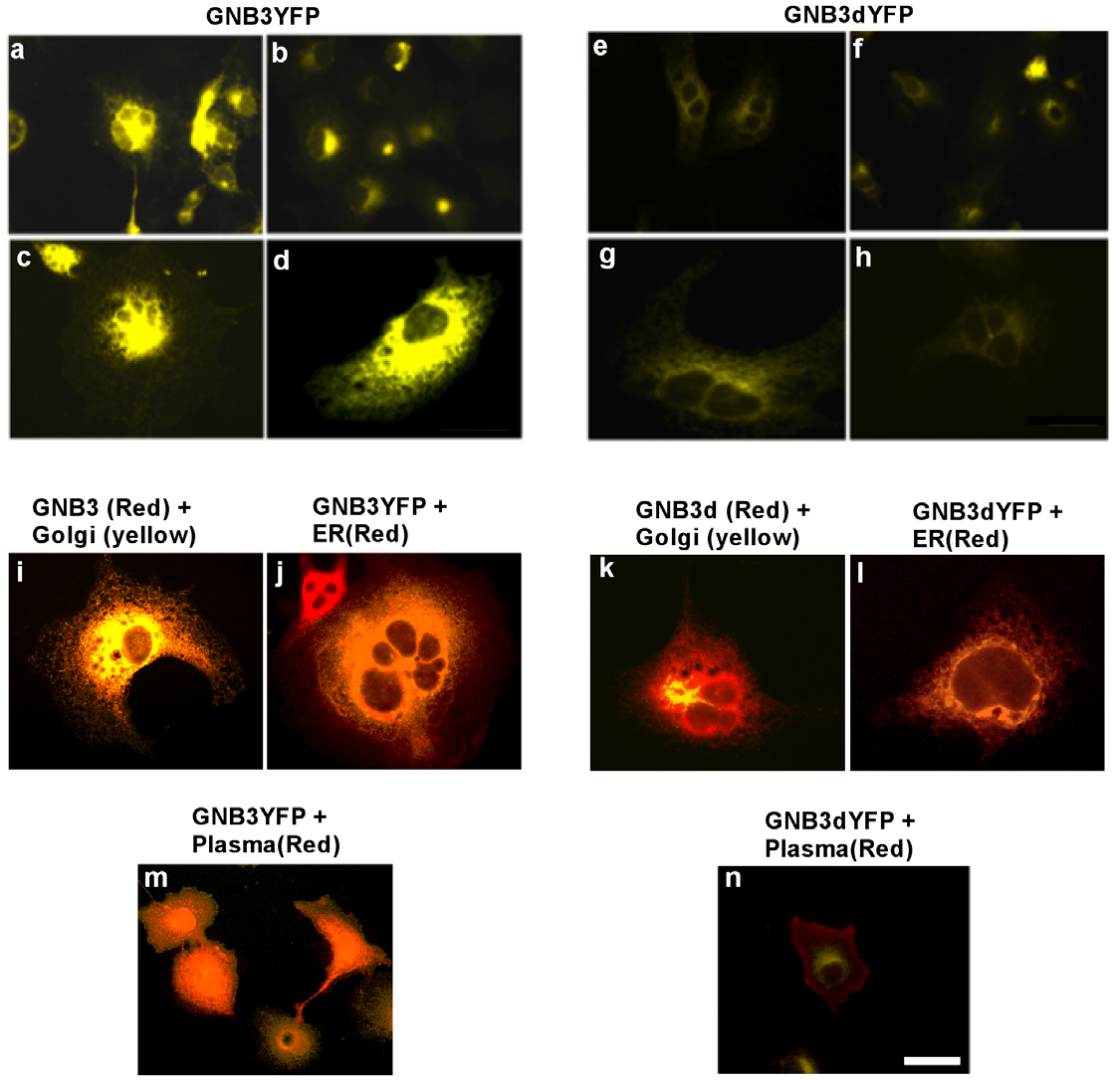
Differential localisation of normal and mutant GNB3 proteins. COS-7 cells transiently transfected with recombinant constructs expressing GNB3YFP (Fig. 1a–d) and GNB3dYFP (Fig. 1e–h). Cells expressing GNB3 show ER and Golgi, localisation (1i & j). Cells expressing GNB3d mutant protein show predominant localisation to ER (Fig. 1k) but not in Golgi (Fig. 1l). Plasma membrane staining revealed no colocalisation of GNB3dYFP (Fig. 1n) when compared to GNB3YFP (Fig. 1m). These are representative images taken from different fields of view under 40× objective with 1.5× further magnification. Bar = 8 µm.

**Figure 2:**
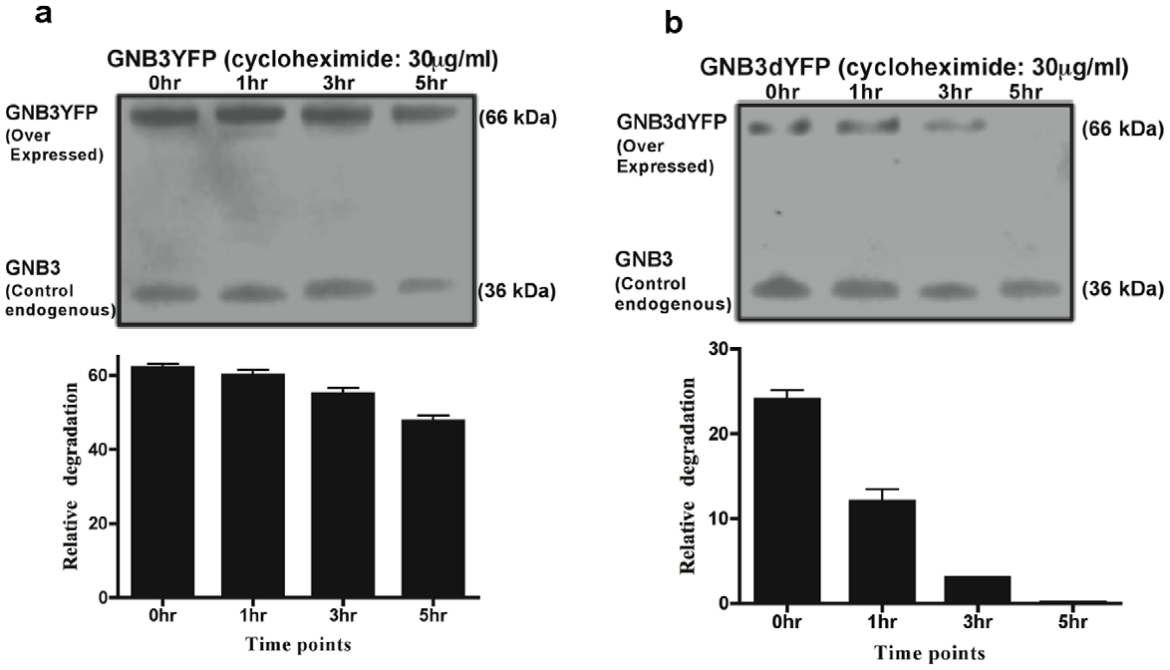
GNB3 degradation assay. Relative degradation of GNB3YFP and GNB3dYFP in COS7 cells at different time points after treatment with cyclohexamide and probed against custom raised GNB3 antibody. The graphs are representative of a total of three independent experiments and relevant readings at each individual time point were normalised to the densitometry units of endogenous GNB3 on the same blot.

**Figure 3:**
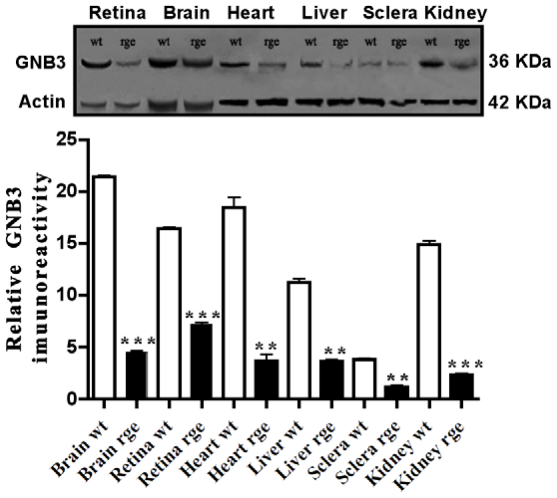
GNB3 protein expression in key tissues of rge chicken. Immunoreactivity of GNB3 on D153del affected (rge) and normal tissue (wt) protein extracts blotted against Anti-GNB3 followed by quantitative densitometry analyses in the D153del affected pooled tissue samples (n = 8) of the same age. Data presented in all panels are the mean with standard error (bars) of n = 3 independent experiments. Statistical significance is shown as * P<0.05, **P<0.01, ***P<0.001.

The presence of the D153del mutation in GNB3 gene homozygous rge birds is predicted to cause an absence of β sheets in propellers 1 and 5 of the GNB protein [30]. This structurally abnormal protein is probably misfolded and targeted to early degradation by the cells ubiquitin proteasome system [36]. Differential expression levels in the subcellular compartments was observed in the case of GNB3dYFP (Fig. 1k-1) when compared to normal GNB3YFP (Fig. 1a–d) in transiently tranfected COS-7 cells. Predominant expression of mutant GNB3dYFP protein is exclusively in the endoplasmic reticulum (ER) compartment (Fig. 1l) when compared to GNB3YFP (Fig. 1f). GNB3YFP protein also shows much more localisation to Golgi (Fig. 1i) and plasma membrane (Fig. 1m) compared to the expression pattern of GNB3dYFP (Fig. 1k & 1n). Blocking protein translation by CHX revealed that GNB3dYFP degrades at a much faster rate than GNB3YFP protein and also when compared to the endogenous GNB3 (Fig. 2a) in COS7 cells. Comparative and relative degradation rate densitometry analysis was performed between GNB3YFP and GNB3dYFP against endogenous GNB3 immuno-blot signals, and showed that mutant GNB3dYFP degrades in less than 5 hours (Fig. 2b). As the GNB3d protein is localised predominantly in ER (Fig. 1e) it is probably destined for degradation through the ubiquitin conjugated proteosomal pathway much faster than normal GNB3. As expected in rge chicken tissues carrying the D153del mutation in GNB3 gene, significant fold decreases in GNB3 immuno-reactivity were observed in all the rge chicken tissues studied in comparison to wt (Fig. 3).

### Loss of GNB3 protein causes differential regulation of cyclic nucleotide secondary messengers

**Figure 4:**
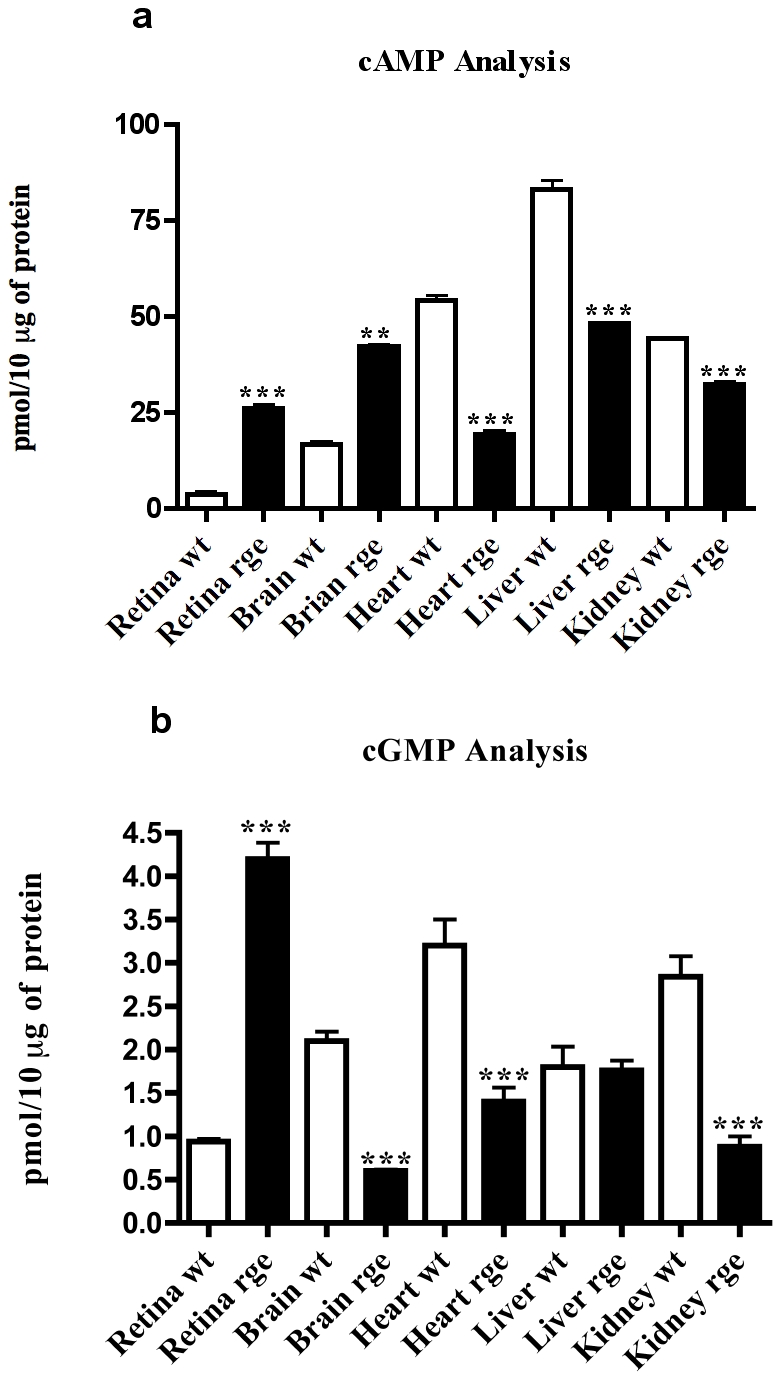
Differential regulation of cGMP and cAMP in rge chicken tissues. **a**) Tissue specific regulation of cGMP levels are shown by a significant fold increase in D153del affected retina, but decrease in other D153del tissues compared to unaffected controls. Data are the means and standard error (bars) of n = 3 independent experiments performed in octuplets. Statistical significance is shown as * P<0.05, **P<0.01, ***P<0.001. **b**) Tissue specific regulation of cAMP levels are, shown by a significant fold increase in D153del affected retina and brain samples and decreased in D153del affected heart, liver and kidney, when compared to levels of normal controls. Data are the mean with standard error (bars) of n = 3 independent experiments performed in octuplets. Statistical significance is shown as * P<0.05, **P<0.01, ***P<0.001.

Both cGMP and cAMP play a crucial role as key mediators of intracellular signaling regulated by the Gβγ pathway that controls various biochemical events. For example cyclic nucleotide levels are altered significantly in other animal models possessing recessively inherited visual transduction mutations [Bibr pone.0021156-Fox1] [Bibr pone.0021156-Davis1] [37–39]. Retinal tissues from homozygous rge birds showed altered levels of cGMP regulation compared with wild type controls (Fig. 4b). Retinal tissues from homozygous rge birds showed a significant fold increase in cGMP and cAMP when compared to normal wt controls (Fig. 4a & Fig. 4b). In contrast rge brain, heart, and kidney tissues, showed a significant fold decrease in cGMP concentration when compared to wt controls (Fig. 4b). Moreover up regulation of cAMP levels in rge brain tissue was observed compared to down regulation of cAMP in rge heart, liver and kidney tissue extracts against their normal wt age matched controls (Fig. 4b). These observed effects in rge chicken tissues suggests that the GNB3 subunit has a tissue specific role in maintaining cyclic nucleotide levels.

### Mutant GNB3 subunit mediates aberrant phosphosignaling activities in rge chicken tissues

**Figure 5:**
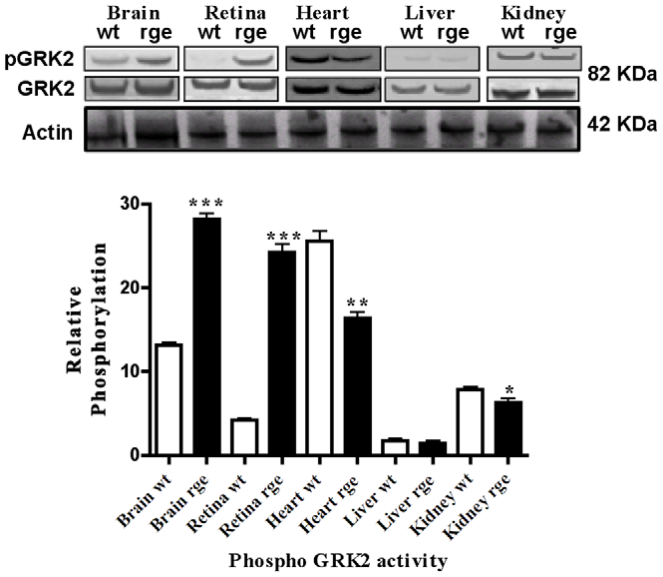
GNB3 controlled cAMP regulates GRK2 phosphorylation activity. Phospho immuno reactivity of GRK2 in D153del affected retina, brain, heart liver and kidney show tissue specific changes in rge chicken protein extracts compared to wt controls. Experiments represent a total of n = 3 independent experiments. Statistical significance is shown as * P<0.05, **P<0.01, ***P<0.001.

**Figure 6:**
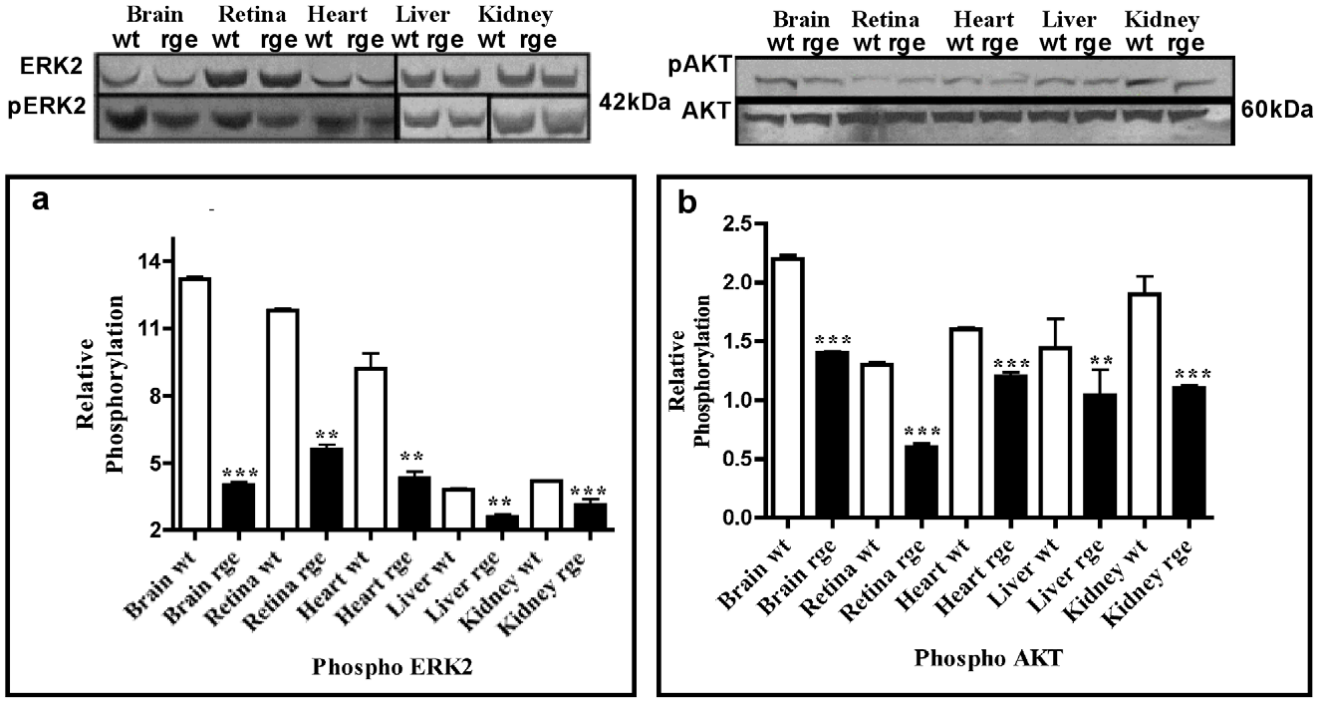
Lack of ERK2 and AKT phosphorylation. Phospho immuno reactivity of ERK2 and AKT in D153del affected retina, brain, heart liver and kidney. Mutant *rge* chicken protein extracts showed significant changes in relative phosphorylation activity when compared to normal age matched controls that was confirmed by semi quantitative densitometry analyses. Experiments represent a total of n = 3 independent experiment. Statistical significance is shown as * P<0.05, **P<0.01, ***P<0.001.

Cyclic AMP exerts its effects in controlling most aspects of cellular signaling. Altered level of cAMP regulation can actually orchestrate differential regulation of the microenvironment by recruiting a variety of signaling and scaffolding molecules. Tissue specific phosphorylation of GRK2, observed in the mutant GNB3 tissues (Fig. 5), are associated with the regulating cAMP levels. This suggests that Gβγ controlled AC's, which synthesize cAMP are primarily involved in phosphor regulation of GRK2 as well as downstream pathways that involve AKT and MAPK. A decrease in phospho ERK2 and AKT activity was also observed in rge tissues (P<0.001) when compared to wt controls (Fig. 6a & 6b). Gβγ activates AKT in PI3K dependent fashion and has been implicated in playing a role in cell growth survival and proliferation [40]. The mutant GNB3 subunit mediated changes in phosphorylation activity strongly suggest that the reduced signaling patterns are indeed post translational regulatory defects, as the total levels of ERK2 and AKT showed no difference in rge chicken tissues when compared to wt controls (Fig. 6a & 6b). The reduced phospho ERK2 activity suggests that rge tissues suffer from both defective translocation and phosphorylation of its target proteins to multiple cell compartments even after stimulus in the signal transduction process.

### rge chickens suffer from renal disease

**Figure 7:**
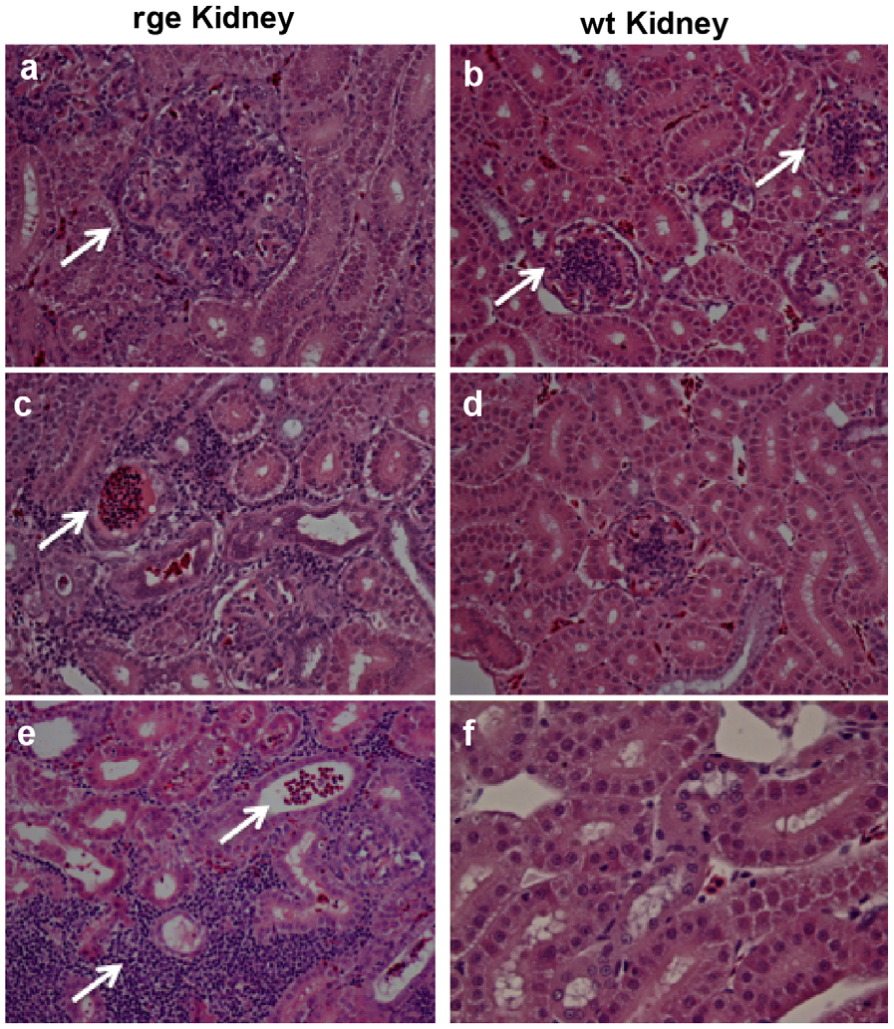
Histopathological findings on kidney sections of rge chickens. Glomeruli from mutant kidneys (Fig. 7a) are considerably larger than their wild type counterparts (Fig. 7b); whereas tubules are of approximately the same diameter. Red blood cells in large numbers are seen in the lumen of tubules and where present are associated with flattening injury to the tubular epithelium (Fig. 7c and d). These red cells are not seen in wild type kidneys (Fig. 7b, d, & f). There is a focus of severe tubulo-interstitial inflammation (lower arrow) comprising lymphocytes and macrophages infiltrating tubules in rge that are not seen in wt kidneys. Magnification 66×.

**Figure 8:**
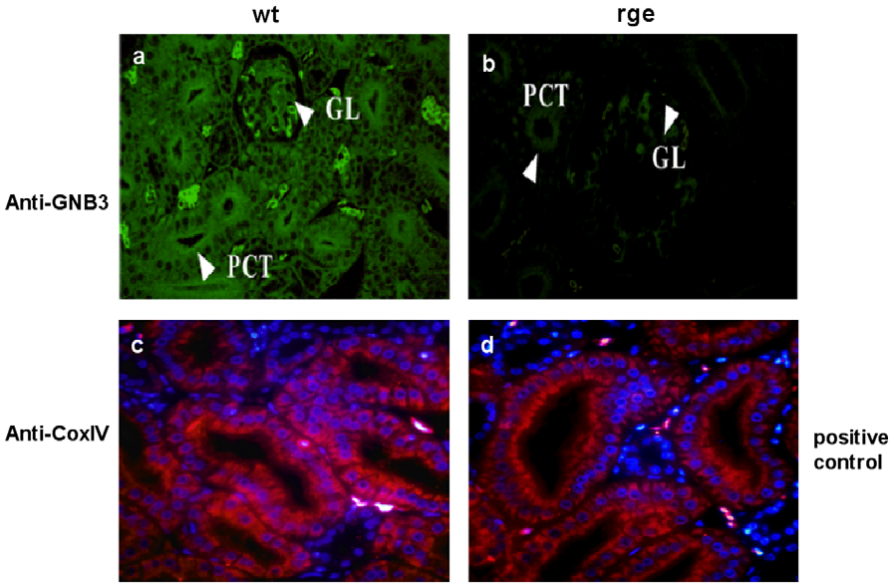
Predominant GNB3 expression in wt kidney sections. Immunohistochemistry of GNB3 (green) on kidney sections shows high expression in the epithelial border of the tubules, proximal convoluted tubule (PCT) and glomeruli (GL) of normal kidneys (Fig. 8a), which are less evident in rge sections (Fig. 8b). Postive control staining of COX IV antigen (red) is observed in both rge and wt sections (Fig. 8c & 8d). Figures are representative of different images taken from different fields of view in three individual experiments (n = 3). Magnification 60×.

Histological examination of haematoxylin and eosin stained sections showed significant renal pathology in mutant birds compared to wild type controls. For example it is clearly evident that rge birds suffer from glomerulopathy as the sections show enhanced glomerular size (Fig. 7a) at the same magnification (60×) in comparison to wt kidney sections (Fig. 7b). Red blood cells were noted in the urinary filtration space of glomeruli (Fig. 7c). Abundant red blood cells were also seen in tubular lumina, and tubular injury with flattening of the tubular epithelium was associated with red blood cells in rge kidney sections (Fig. 7c) but not in wt (Fig. 7d). There was a marked focal tubulo-interstitial inflammatory infiltrate, with lymphocytes and macrophages infiltrating tubules in rge kidney sections (Fig. 7e). In some, but not all, foci neutrophils were present in rge sections (Fig. 7e) but not in wt (Fig. 7f). GNB3 immunohisto reactivity was totally diminished in the renal cells of the rge birds compared with an overt expression pattern of GNB3 in proximal convoluted tubule (PCT) and glomerulus (GL) in wt sections (Fig. 8a). CoxIV, a mitochondrial protein, was present in both wt and rge sections confirming the reliability of the IHC technique (Fig. 8b). The GNB3 immuno reactivity results are consistent with our previous findings that the D153del mutation affected GNB3 protein structure [30], stability, and cellular localisation (Fig. 1) with much shorter half-life than the normal GNB3 protein, as shown in our present degradation studies (Fig. 2). Given the prominent GNB3 expression in rge kidney sections the related pathological finding observed in rge chickens are considered to be a primary genetic defect causing loss of function of a specific renal transport protein or signaling molecule. Consequently, the functional disturbances of certain tubule segments lead to defects in tubular reabsorption (Fig. 7c & e). The changes of GNB3 protein expression in whole kidney observed by Western blot likely reflect GNB3 expression, as observed in the IHC.

## Discussion

### Role of GNB3 protein signaling in photoactivation of cone cells

Our results indicate that the D153del mutation results in an unstable GNB3d protein. This structurally abnormal protein is probably misfolded and targeted for early degradation by the cells ubiquitin proteasome system [36], when compared to the normal GNB3 protein. The lack of GNB3 protein in the cell machinery will probably inhibit trafficking and tethering of the Gα subunit to the plasma membrane [41] and coatomer binding to the Golgi membranes [42]. Recent studies on Gβγ signalling in endomembranes of the cell have implied a role in protein transport through the trans Golgi network (TGN) [43]. Normal GNB3YFP appears to be colocalised with Golgi vesicular stacked structures as observed in Fig. 2c, while this colocalisation pattern is absent in the mutant GNB3dYFP. As seen in Fig. 2d GNB3dYFP is mislocalised and retained in ER colocalising with calreticlin ER marker (red). The GNB3dYFP appears to be unable to traffic to the Golgi and other cell compartments. In contrast, as normal GNB3YFP is expressed in a Golgi, this implies that most of its intracellular signalling includes the vesicle formation pathway between the TGN and the plasma membrane [43]. The deleterious impact of D153del mutation on GNB3 structure and its localization might suggest that it is very unlikely to form stable heterotrimers and heterodimers. Therefore the mutant GNB3dYFP is no longer considered as a *bona fide* resident in the ER and will probably be targeted for early degradation through the ubiquitin conjugated proteosomal pathway (Fig. 2b). In the normal visual transduction pathway, the Gα transducin subunit 2 (G_t_α2), is responsible for activating phosphodiesterases (PDE6b) that hydrolyzes the synthesized cGMP from Guanate Cyclases (GC's). Therefore any decrease in PDE6b activation will result in an increase of cGMP due to less cleavage of these molecules. Moreover the lack of stable interaction with both the G_t_α2 transducin and Gγ subunits will lead to the loss of photoactivation in cone cells. cGMP is a common regulator of ion channel conductance, glycogenolysis, and cellular apoptosis and also participates in synaptic signaling and neuronal cell physiology. Any alterations in cGMP levels may also change brain functional physiology [Bibr pone.0021156-Fiscus1] [44,45]. Insufficient cGMP levels observed in whole brain tissue suggests that cGMP-mediated pathways involving cGMP dependant gated ion channels, cGMP dependent kinases and cGMP controlled PDE's as generators, effectors and modulators of neuronal development and function [Bibr pone.0021156-Fiscus1] [44,46], are likely to be affected. The increase of cGMP has previously been shown to cause a continuous opening of cGMP dependant ion channels and lead to a drastically elevated Na^+^ and Ca^2+^ flux [47]. The extremely elevated ion levels may contribute primarily to disturbance in vision and secondarily, result in retinal dystrophies [Bibr pone.0021156-Ionita1] [47,48]. As rge photoreceptors remain intact but become increasingly disorganized [31], it is possible that significant alterations in the expression of connexin proteins, which are observed in the PDE6b rd mouse, may also be occurring [49]. G_t_β2 is also known to inhibit ACs [50] and due to its lower activation results in less or no transport to the membrane and inhibits ACs. Therefore high increases in cAMP levels generated in the rge eye suggests that they are likely to have fewer other activated alpha transducin subunits that bind to the unstable GNB3 subunit (Fig. 4b). High local levels of cAMP can be toxic to photoreceptor cells which become unresponsive to survival factors, due to altered signaling [47], and this may ultimately contribute to retinal dysfunction causing the cone cell disorganisation as observed in rge birds [31]. Increased levels of cAMP can also activate protein kinases that are coupled to G proteins, especially GRK2. Increase in relative phosphorylation levels of GRK2 under basal conditions observed in rge retina is therefore due to an increase in the level of cAMP (Fig. 4b & Fig. 5). This suggests that the likely mechanism altering the desensitization kinetics of associated GPCR is due to the lack of both translocation and the binding of upregulated phospho GRK2 to the Gβγ subunits at the plasma membrane. The lack of phosphorylation of ERK2 and AKT in the rge eye also suggests that an inadequate response generated through deregulated signaling of GPCR due to the mutant GNB3d subunit failing to translocate to the plasma membrane (Fig. 1 & 6).

### Role of Gβγ signaling in G_s_ and G_i/o_ pathways

The differential regulation of GNB3 signaling associated with tissue specificity, can be explained in relation to the Adenylyl Cyclase (AC)'s isoforms, which are primarly activated and controlled by Gβγ in the GPCR pathway. Nine different isoforms of AC exist and can significantly contribute to the diversity of cellular cAMP concentration. Gβγ exerts its effects on particular AC isoforms, which can be either negatively (ACI) or positively regulated (ACII, ACIV & ACVII) [Bibr pone.0021156-Bommakanti1] [40,41]. For example, in the brain cells of the rge chicken there could be no inhibition of type I ACs due to an unstable GNB3 subunit signaling complex, leading to constitutive accumulation of cAMP (Fig. 3b). In contrast, chicken heart, liver and kidney samples probably have a preponderance of cells which express positively regulated ACII, ACIV & ACVII compared to ACI resulting in constitutive deregulation of cAMP levels in these tissues (Fig. 4b). Most importantly, G protein signalling pathways have been characterised in either G_s_/G_i/o_ depending upon the type of activity they generate in the intracellular signalling cascade [1]. Tissue specific signaling exerted by GNB3 subunits suggests that their effects are most likely due to differences in the presence or absence of G_s_ or G_i/o_ pathways in different cell types. For example in the chicken brain, cells which likely activate the G_i/o_ pathway, will have an inhibitory effect on type I Adenylate Cyclase (AC), that probably outnumber cells that activate the G_s_ pathway. In rge eye and brain, the G_i/o_ pathway is likely to be activated, due to the unstable mutant GNB3 protein, causing less inhibition of type I AC, leading to an increase in cAMP in these tissues (Fig. 3). In contrast, chicken heart, liver and kidney samples probably have a preponderance of cells which activate the G_s_ pathway compared to cells that activate G_i/o_ leading to a decrease in cAMP levels in these tissues (Fig. 3). The Gβγ complex of heterotrimeric G proteins is the most studied molecule for the divergent regulation of ACs that is activated or inhibited respectively through G_s_ and G_i/o_ pathway. The tissue specific regulation in the rge birds, suggest the vital importance of the GNB3 subunit in exerting differential effects involving ACs in relation to G_s_ or G_i/o_ pathways. Distinct regulation in phospho GRK2 in rge tissues might cause differential activation in translocation of GRK2 molecules to the plasma membrane thereby causing disregulation in desensitization kinetics of the associated GPCR. Moreover ERK1/2 molecules mediate principal signaling cascades that transmit signals from cell surface receptors to cytoplasmic and nuclear effectors. Loss of phospho ERK2 and AKT activity which are immediate effectors of PI3K and RAS pathways suggests that the mutant GNB3 subunit has lost its control over these pathways in rge chicken tissues (Fig. 6a & b). As a consequence, discrepancies in intracellular molecular signaling occurred in rge tissues (Fig. 5, 6a & b) that fail to stimulate these subunits in a dual and complementary way, i.e., by recruitment and activation at membrane regions as observed previously in *in vivo* studies [56].

### Understanding the pleitropic effects of Gβγ signaling ‘clue to complex syndromes’

A previous screen of the human GNB3 gene for mutations in 164 patients with non-syndromic cone-rod or macular dystrophy revealed no putative pathogenic mutations [51]. This result is likely to be due to the ubiquitously expressed GNB3 protein, which will almost certainly produce similar pleiotropic signalling and pathological effects as seen in the rge chicken. For example the prominent expression pattern of GNB3 in renal section of normal chicken (Fig. 3a & b) suggests that it might be involved in the principal functions of an organ that are controlled by phosphorylation and intracellular trafficking. Humans homozygous for severely mutated GNB3 genes are therefore more likely to suffer from a complex syndromic disorder e.g. cone-rod dystrophy or achromatopsia, with a kidney and possibly other abnormalities in other key tissues.

Similarly 825TT humans expressing the GNB3s protein subunit, will probably show a general increase in cAMP levels and phospho proteome in kidney cells, due to an enhanced signaling effect, when compared to 825CC individuals. An increase in cAMP levels in the kidney cortical collecting duct cells would have the effect of altering the expression of channels such as aquaporin or epithelial sodium channels (ENaC) [52]. A subtle alteration in the expression of these channels would lead to alterations in the salt concentrations of blood. This in turn could be one of the underlying reasons why GNB3 825T individuals are predisposed to hypertension.

The relative levels of cyclic nucleotides and phospho regulation in this study reveal the molecular basis of retinal disease pathogenesis. Glomerulomegaly and tubulo interstitial inflammatory lesions observed in rge kidneys have now been related to the irregular signaling patterns caused by the D153del GNB3 mutation in our present study. Thus we speculate that these novel pathology findings in rge chicken kidneys (Fig. 7a & 1c) resemble symptoms of obesity related glomerulopathy characterized as glomerulomegaly in humans [Bibr pone.0021156-Kambham1] [53,55]. In humans this disease remains one of the most intractable kidney diseases and mostly in children it carries a 30% risk of recurrence in a kidney transplant [54]. The renal tubular inflammation (Fig. 7c) and glomerular infiltrate of blood cells (Fig. 7e) observed in rge chicken kidneys might be due to lack of proper renal tubular function that governs reabsortion of water and solutes from the glomerular filtrate. An increasing number of tubulopathies are being recognized as caused by single-gene mutations [54]. As mutation 825C>T in the GNB3 gene is well studied in worldwide population in regards to complex diseases [Bibr pone.0021156-Siffert1] [Bibr pone.0021156-Weinstein3] [Bibr pone.0021156-Siffert2] [21, 22, 24, & 25], it is now evident that it is of prime importance that these alleles are viewed in the context of “*personalized medicine*”. The 825T allele conveys a significant increase in risk for both homozygote and heterozygote humans to develop a disease in due course [Bibr pone.0021156-Weinstein1] [4,56].

In conclusion the present study indicates that the rge chicken is a valuable animal model for similar renal and eye phenotype disease studies. The mutant GNB3 subunit and its subsequent effects on phosphorylation signaling pathways can be suggestive of biomarkers for the identified pathologies of relevance to human beings. Although aberrant signaling regulation was observed in brain, heart, and liver rge tissues no pathology has been identified or characterised in the rge chickens (Figs.S1, S2, S3, S4) implying a tissue specific role of GNB3 in defining pathogenesis.

## Materials and Methods

### Chickens, H&E staining and immuno histochemistry (IHC)

#### Ethics statement

The rge chicken lines were maintained at the Roslin Institute, as described [57] under a UK Home Office project license. All animals were treated in accordance with the UK Home Office regulations for the use of animals in research. Retina, brain, heart, liver and kidney tissue samples were isolated from normal and rge affected chickens (n = 8) of the same age for tissue section (8 months old). For H&E staining and IHC, sections were cut at 4 µm thickness using microtome blade (35°/80 mm, Shandon, UK) that was equipped to the microtome (Shardon Retraction AS325, UK). Subsequently all sections were fixed in 10% buffered neutral formalin and embedded in paraffin sections. All of these sections were stained with hematoxylin and eosin (H&E) and observed under 60× magnification using Leica comparison microscopy systems. For immunohistochemistry paraffin embedded sections were deparaffinised and incubated in 3% H2O2 to block endogenous peroxidase activity. Sections were further subjected to epitope un-masking by a heat induced epitope retrieval system to avoid the cross linking of the proteins formed by formalin fixation. Sections were blocked using 5% goat serum in PBS to completely avoid the background staining. Primary antibody incubations were carried out for 2 days at 4°C using custom raised anti-rabbit GNB3 primary antibody diluted in blocking solution (1∶500), subsequently washed and then treated with fluorescein isothiocyanate (FITC)-conjugated (Sigma cat no: F9887) antirabbit IgG (1∶100). For positive control CoxIV antibody (Abcam Biosciences cat no: 33985) that detects mitochondrial protein in tubules of kidney is been used at 1∶200 dilution in blocking solution, subsequently washed and then treated with Alexa Fluor 568-conjugated (Invitrogen) goat antibody anti-mouse IgG (1∶250). Slices were washed, mounted with vectashield mounting reagent (Vector Laboratories, Peterborough UK) on a cover glass, and subjected to imaging using fluorescence Leica DMire2 microscope under 40× magnification for GNB3 and positive control CoxIV antigen. Images were collected using excitation and bandpass filters optimal for FITC (GNB3), Texas red (CoxIV) and UV (DAPI) staining.

### Cell culture, plasmids, transfections and treatments

Simian COS-7 (ATCC CRL 1651) cells were maintained in DMEM (Invitrogen, UK) supplemented with 10% foetal bovine serum (FBS), 2 mM glutamine, 1 mM sodium pyruvate, 100 µg/ml streptomycin and 100 U/ml penicillin in an atmosphere of 5% CO2. Prep4GNB3 construct was a kind gift from Dr M.J. Bullido (Universidad Autónoma de Madrid, Cantoblanco, Madrid, Spain). GNB3YFP construct was achieved by PCR of YFP from a recombinant construct given as kind gift from Prof. David J. Chen (UT Southwestern Medical Center, Texas, USA) using primers carrying flanking restriction sites forward *KpnI* 5′ *CCAGGTACCATGGTGAGCAAGGGCGAGGAGCTGTTCACCGGGG3′* and reverse *HindIII* *5′AAGCTTCTTGTACAGCTCGTCCATGCCGAGAGT3′* . The final PCR product was digested and subcloned into the multiple cloning site of linearised Prep4GNB3 constructs upstream of the GNB3 gene between *KpnI* and *HindIII* sites to create N terminal fusion of YFP into the MCS of digested Prep4GNB3 construct with *KpnI* and *HindIII* restriction enymes, to create the GNB3YFP construct. Several groups have shown N-terminal FP tags of the G protein β and γ subunits to be functionally active [58]. The GNB3dYFP construct was generated by a mutagenesis approach with the QuickChange II Site Directed Mutagenesis Kit (Stratagene, UK) using primers forward *5′ CTCTCCTGCTGCCGGTTTCTAGACAACAGTATTGTG3′* and reverse *5′CACAATACTGTTGTCTAGAAACCGGCAGCAGGAGAG3′* . The fidelity of the constructs have been checked and verified by sequencing using commercial sequencing service (www.dnaseq.co.uk). Transfection of these constructs was performed on exponentially growing COS-7 cells by electroporation (400 V, 350 µF using Easyject EquiBio, Kent, UK). 10 µg of DNA was used per transfection (2×10^6^ cells) and 16 to 24 hr post transfection cells were processed for further analysis. Protein half-life studies in COS7 cells has been performed by transfecting 10 µg of GNB3YFP and GNB3dYFP DNA constructs and subjected to cycloheximide (CHX)(Cat No: C104450, Sigma Aldrich, UK) treatment 30 µg/ml in DMEM medium until relevant time points to inhibit protein synthesis.

### Imaging and Colocalisation

Transfected COS-7 cells with YFP tagged plasmid constructs were grown in standard glass coverslips coated with polylysine (sigma P8920) placed in petridishes containing Dulbecco's modified Eagle's medium supplemented with 10% fetal bovine serum. 16 h post transfection cells were fixed in 3% paraformaldehyde in a standard PBS at room temperature for 30 min. The cells were gently washed twice with 1 ml of PBS, blocked by 1% goat serum, 1% bovine serum albumin in PBS containing 0.05% Triton X-100, for 30 min, incubated with anti-rabbit calreticulin antibody (Abcam cat no: ab 4) for ER staining in PBS, 0.3% Triton X-100 for 30 min, washed three times with the 0.3% Triton X-100/PBS for 5 min, and then incubated with Alexa Fluor 568-conjugated goat anti-rabbit antibody (Invitrogen) for 30 min. For plasma membrane staining CellMask™ Deep Red stain from Invitrogen (C10046) was used on transfected cells at 2.5 µg/ml concentration in PBS for 1 hr before fixation. For Golgi colocalisation cells were transfected with untagged GNB3 and GNB3d plasmid along with pEYFP-Golgi purchased from Clontech labs. The cells mounted with Vectashield solution (cat no: H-1000; Vector Laboratories, Peterborough, UK) and imaged under Leica DMiRe2 electronic microscope. Images were collected under relevant excitation and emission filters depending on the fluorotype using an iXonEM +897 EMCCD camera (ANDOR Technologies Ltd, USA) and visualised using multi dimensional microscopy software Andor Module iQ Core. Colocalisation assays were performed and determined with software integral features supplied by Andor IQ core software features.

### Protein extraction and quantification

Retina, brain, heart, liver and kidney tissue samples were isolated from normal and rge affected chickens (n = 8) of different ages and snap frozen in liquid nitrogen before storage at −80°C. Thawed tissues of similar age matched White Leghorn controls along with rge tissues (n = 8) were pooled together, weighed and placed in tissue extraction buffer (RIPA Pierce Biotech) containing a protease and phosphatase inhibitor cocktail (Pierce Biotech 100×). All the protein extracts were prepared as in [12] except that heart, liver and kidney protein extracts were subjected to sonication of 2 cycles for 10 seconds at 50% pulse. The final mixture was shaken gently on ice for 15 min and the protein supernatant was obtained by centrifuging the lysates at 14000 g for 15 min. For protein half-life studies transfected COS-7 cells were washed in ice-cold PBS and subsequently extracted using 0.01% NP-40 detergent in PBS containing cocktail of protease and phosphotase inhibitors. Cells were centrifuged at 13000 rpm to spin down nuclei and supernatant containing cytosolic fractions and processed for further analysis. Subsequently all protein extracts were quantified using the Bradford reagent against BSA as a standard.

### Antibodies and Immunoblotting

Anti-GNB3 serum was custom raised in rabbit against the peptide sequence ‘MGEMEQMKQEA+C’, that recognises the amino-terminal end of chicken GNB3 (Eurogentec S.A., Liege, Belgium). The cysteine residue (+C) was added to the peptide sequence for conjugation to the carrier protein, keyhole limpet haemocyanin. The sera were affinity-purified against the peptide prior to use. The peptide sequence raised has affinity to cross react with other species such as human, mice, and monkey as the GNB3 gene is evolutionary conserved and posses 100% sequence identity. This antibody might not cross react with other GNB subunits as this peptide sequence is not identical to the N terminal region of the other GNB subunits even though 85% total sequence homology exists between other GNB subunits1-4 but only 40% for GNB5. The GNB3 antibody was used to detect GNB3 expression at relevant dilutions on all chicken tissues and transfected COS-7 cell lines. Expression levels of phosphorylated/total levels of GRK2 (Abcam Biosciences cat no: ab4473/ab81578), ERK2 (Abcam Biosciences cat no: ab50011/ab17942), and AKT proteins (Cell Signaling Technologies Cat no: mAb 2965/9272) were detected by western blotting. Briefly, 20 µg concentration of tissue protein lysates in sample buffer were loaded per lane on 10% SDS–polyacrylamide gels. After electrophoresis, the proteins were transferred to nitrocellulose membranes (Bio-Rad Hybond ECL) using the Invitrogen XCell SureLock Mini-Cell system and processed using a commercially available kit (WesternBreeze™ Chromogenic Detection Kit, Invitrogen. cat no: WB7105, WB7103). Non-specific reactivity was blocked by incubation with the blocking reagent supplied in the kit. Membranes were further processed by incubating with primary antibodies for 2 hours at room temperature or overnight at 4°C, followed by incubation for 1 hour at room temperature with appropriate secondary antibody anti mouse or anti rabbit supplied in the kit. Bands were visualized with the enhanced chromogenic substrate analysis system supplied in the kit. For loading control, the levels of β-actin were examined on the same blot by incubating the blots with stripping solution for one hour (20% SDS, 67.5 µM tris-HCl of a pH 6.7, 100 um 2- Me) and reprobing with anti mouse β-Actin antibody (Abcam Bioscience cat no: ab8227). Semi quantitative analysis of raw immunoblots was performed by capturing the images in high resolution TIFF format files using a charge-coupled-device camera (AxioCam MRc, Carl Zeiss) and subjected to Gelpro analysis software (Gelpro Software, San Diego, USA) for densitometry.

### Cyclic nucleotide assays

Global cAMP and cGMP measurement assays were carried out to detect variations in the concentration levels in the chicken tissues. The protein extracts were acetylated following the kit instructions (Cayman Biosciences cat no: 581001 for cAMP and 581021 for cGMP) to increase the sensitivity of detection. For cyclic nucleotide determination protein samples are diluted with 0.1 M HCL that inactivates phosphodiesterases and lower the concentration of immunoglobulins that may interferes with the assay. Briefly 10 µg/well concentration of protein lysates were loaded on a 96 well plate, which was precoated with cAMP or GMP polyclonal antibody as octuplets. The polyclonal antibody to cAMP or cGMP binds in a competitive manner to the amount of the corresponding cyclic nucloetides in the sample or an alkaline phosphatase molecule, which has cAMP or cGMP covalently attached to it. After a simultaneous incubation at room temperature the excess reagents were washed away and the substrate supplied in the kit was added. After a short incubation time the enzyme reaction was stopped using reagent supplied in the kit, and the resulting yellow colour was read on a colorimetric microplate reader at 405 nm (Anthos HTIII Microplate Reader, Denley Instruments, Billingshurst, UK). The optical density of the bound colour emitted is inversely proportional to the concentration of cAMP or cGMP level in samples, which was calculated according to the instructions supplied in the kit. All results were analysed by oneway ANOVA with *posthoc* Dunnett tests using the GraphPad Prism 3.02 software. Data was generally expressed as mean ± S.E.M. for individual sets of experiments.

## Supporting Information

Figure S1 **Heart H&E staining.** H&E staining of rge and wt heart muscle tissue showed normal mycocardial histology with no difference in purkinjee layer of muscle cells in longitudinal and circular muscles (1a & 1b). Mild but notable difference in the thickening of epicardial layer (arrow 2 indicated) of rge heart in comparison to wt observed (1c & 1d). Magnification 60×.
(JPG)

Figure S2 **Liver H&E staining.** H&E staining and cross sectional view of liver obtained from both rge and wt birds showing empty spaces representing extruded fat droplets (arrow 1) with appearance of hepatocytes (arrow 2) and macrophages (arrow 3) (Fig a & b). Hepatic hilum is included, hepatic artery intima with endothelial lining (Fig c & d arrow 4 indicated). Magnification 60×.
(JPG)

Figure S3 **Pancreas H&E staining.** H&E staining and cross sectional view of pancreas showing Islets of Langerhans containing α and β cells (arrow 1). Pancreatic acini and exocrine cells (arrow 2) observed in rge and wt birds showed no difference. Magnifcation 60×.
(JPG)

Figure S4 **Brain H&E staining.** H & E staining and cross sectional view of cerebellum at ^×^2.5 magnification showing purkinjee cell layer (PCL) and deep nuclei (DN) of wt and rge brain sections (Fig SF4a & b). Hippocampal sections showing dentate gyrus region (DG) at 2.5× magnification in both rge and wt sections (Fig. SF4e & f). Normal architecture of neuronal cells (arrows indicated) in rge as that of wt birds is shown at 40× magnification reveals.
(JPG)
