# Supplementary figures and images for: The D153del Mutation in GNB3 Gene Causes Tissue Specific Signalling Patterns and an Abnormal Renal Morphology in Rge Chickens
Source: PLoS One. 2011 Aug 22;6(8):e21156. doi: 10.1371/journal.pone.0021156 (PMC3159573; doi:10.1371/journal.pone.0021156)

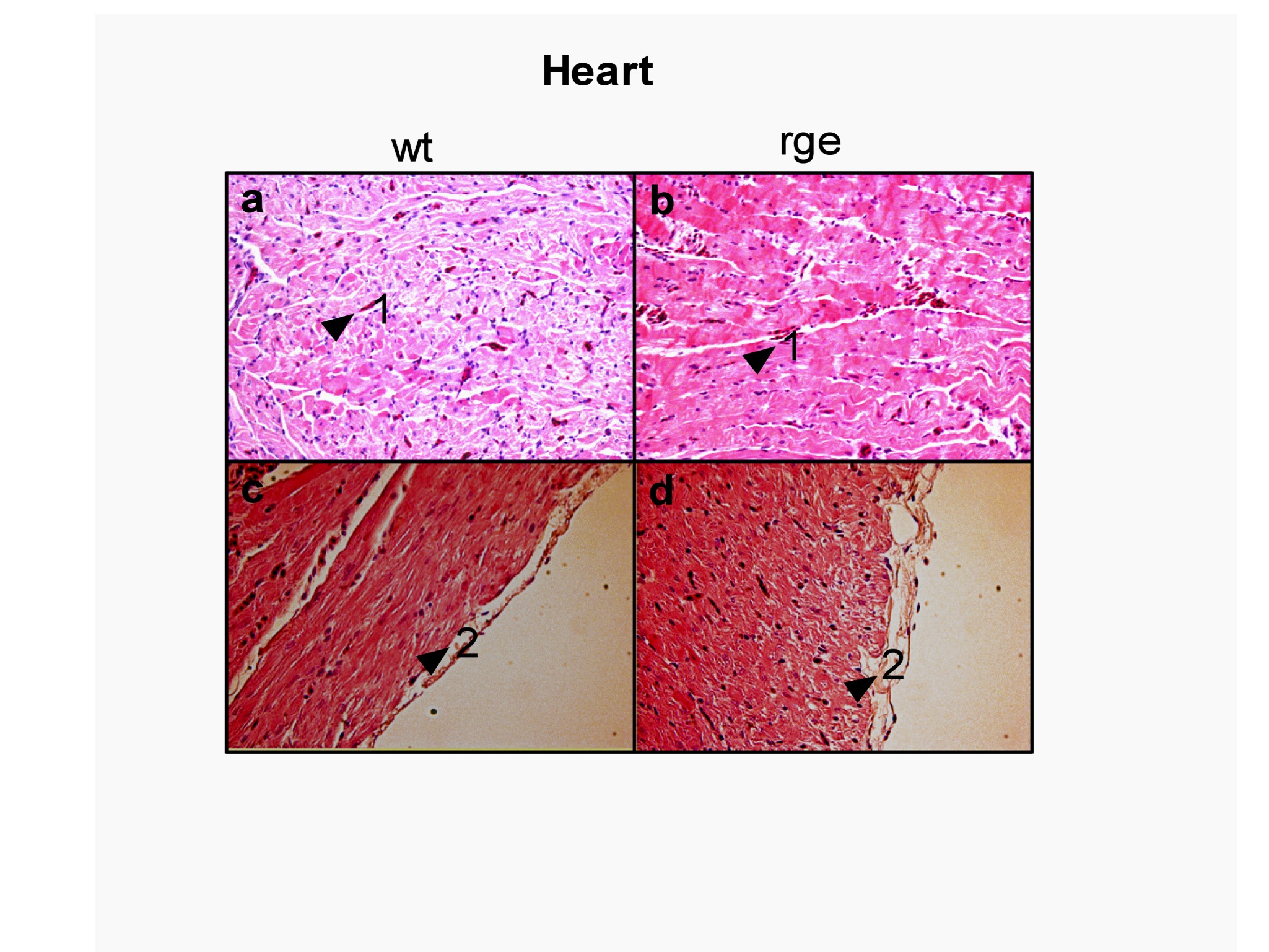

Supplement: Figure S1 — Heart H&E staining. H&E staining of rge and wt heart muscle tissue showed normal mycocardial histology with no difference in purkinjee layer of muscle cells in longitudinal and circular muscles (1a & 1b). Mild but notable difference in the thickening of epicardial layer (arrow 2 indicated) of rge heart in comparison to wt observed (1c & 1d). Magnification 60×. (JPG) [file pone.0021156.s001.jpg]

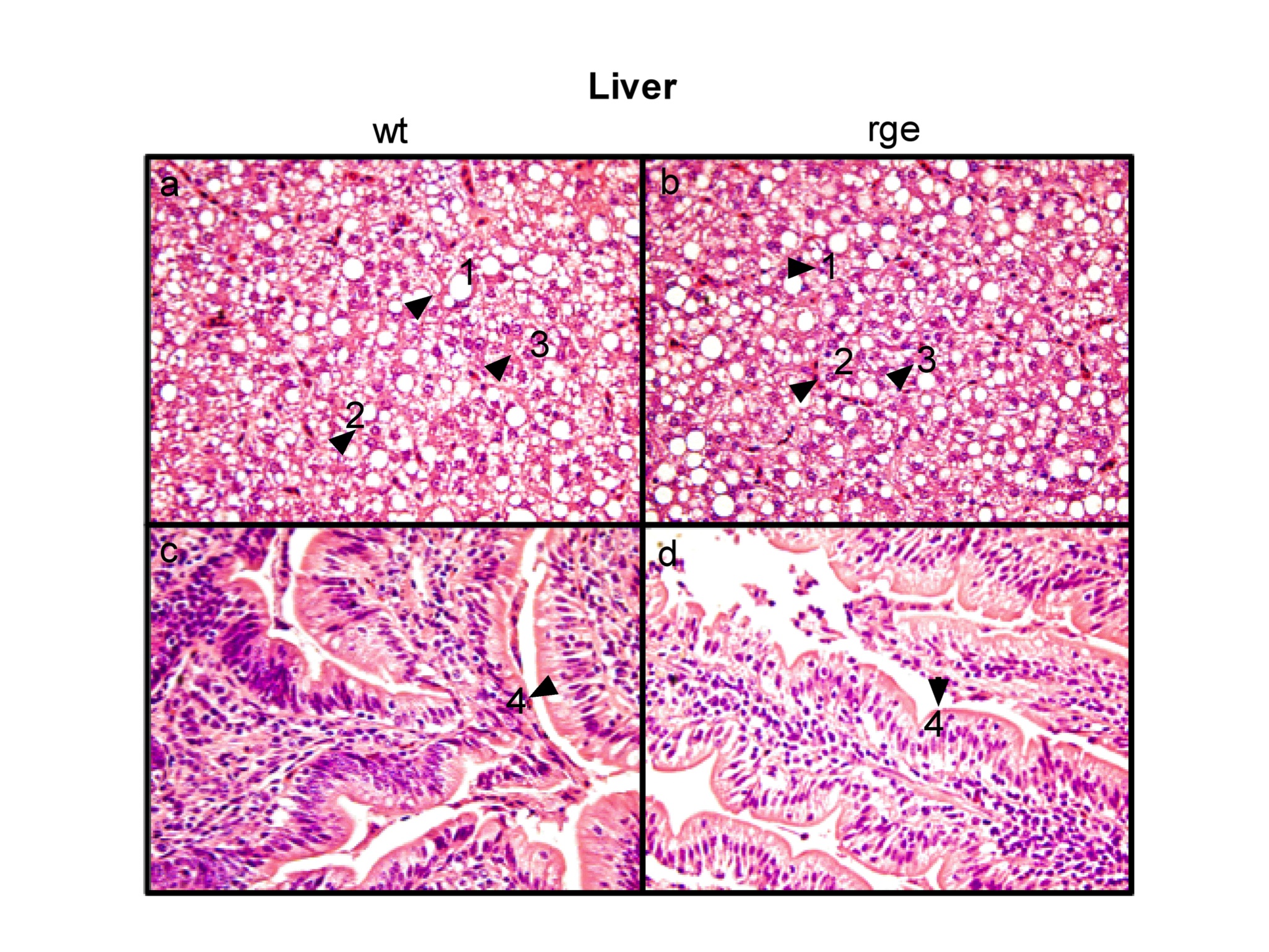

Supplement: Figure S2 — Liver H&E staining. H&E staining and cross sectional view of liver obtained from both rge and wt birds showing empty spaces representing extruded fat droplets (arrow 1) with appearance of hepatocytes (arrow 2) and macrophages (arrow 3) (Fig a & b). Hepatic hilum is included, hepatic artery intima with endothelial lining (Fig c & d arrow 4 indicated). Magnification 60×. (JPG) [file pone.0021156.s002.jpg]

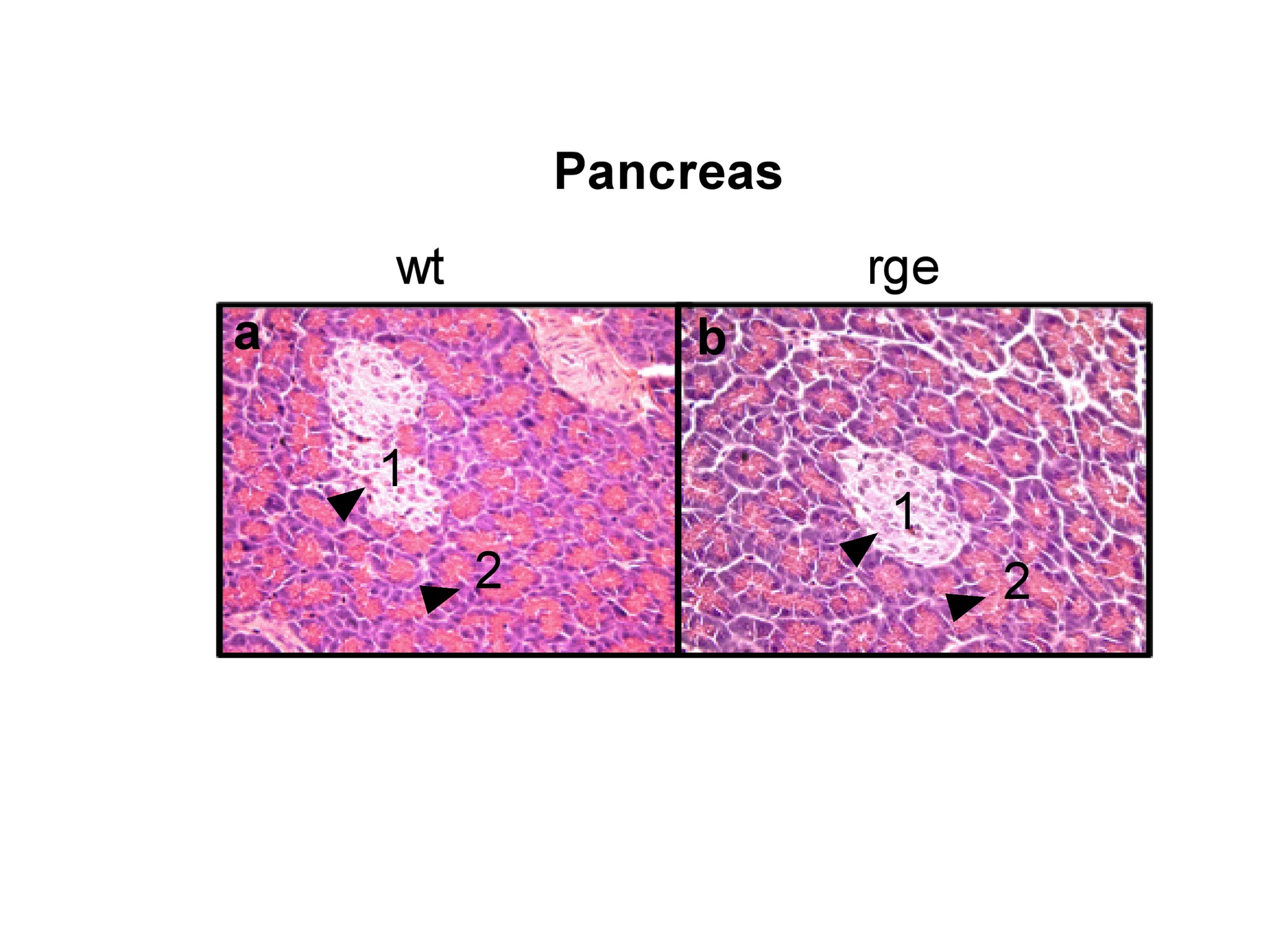

Supplement: Figure S3 — Pancreas H&E staining. H&E staining and cross sectional view of pancreas showing Islets of Langerhans containing α and β cells (arrow 1). Pancreatic acini and exocrine cells (arrow 2) observed in rge and wt birds showed no difference. Magnifcation 60×. (JPG) [file pone.0021156.s003.jpg]

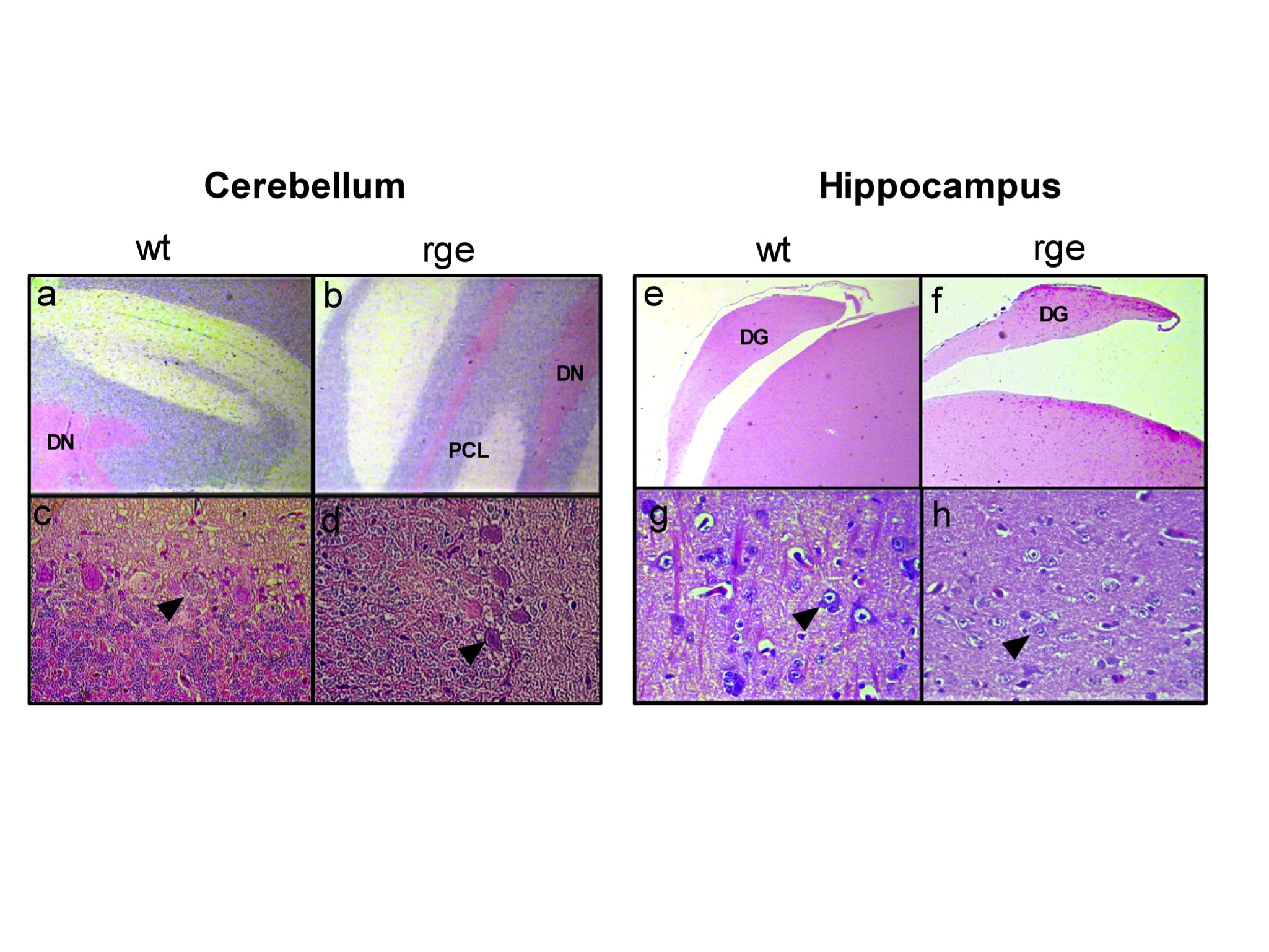

Supplement: Figure S4 — Brain H&E staining. H & E staining and cross sectional view of cerebellum at ×2.5 magnification showing purkinjee cell layer (PCL) and deep nuclei (DN) of wt and rge brain sections (Fig SF4a & b). Hippocampal sections showing dentate gyrus region (DG) at 2.5× magnification in both rge and wt sections (Fig. SF4e & f). Normal architecture of neuronal cells (arrows indicated) in rge as that of wt birds is shown at 40× magnification reveals. (JPG) [file pone.0021156.s004.jpg]
